# Addendum: Resolvins suppress tumor growth and enhance cancer therapy

**DOI:** 10.1084/jem.2017068101232024a

**Published:** 2024-01-31

**Authors:** Megan L. Sulciner, Charles N. Serhan, Molly M. Gilligan, Dayna K. Mudge, Jaimie Chang, Allison Gartung, Kristen A. Lehner, Diane R. Bielenberg, Birgitta Schmidt, Jesmond Dalli, Emily R. Greene, Yael Gus-Brautbar, Julia Piwowarski, Tadanori Mammoto, David Zurakowski, Mauro Perretti, Vikas P. Sukhatme, Arja Kaipainen, Mark W. Kieran, Sui Huang, Dipak Panigrahy

Vol. 215, No. 1 | https://doi.org/10.1084/jem.20170681 | November 30, 2017

After publication, it came to *JEM*’s attention that the article did not include any information for readers to access the raw data of Fig. S3, J and K. These data have now been deposited in the BioStudies database (https://www.ebi.ac.uk/biostudies/) and can be accessed with the accession number S-BSST1262. The HTML version of the article has been edited to include the information about the database and the accession number in the LC-MS-MS section of the Materials and methods. The omission remains in the PDF and print.

